# Unraveling the Regimes of Interfacial Thermal Conductance at a Solid/Liquid Interface

**DOI:** 10.1021/acs.jpcc.4c00536

**Published:** 2024-05-13

**Authors:** Abdullah El-Rifai, Sreehari Perumanath, Matthew K. Borg, Rohit Pillai

**Affiliations:** † Institute for Multiscale Thermofluids, University of Edinburgh, Edinburgh EH9 3FD, U.K.; ‡ Mathematics Institute, University of Warwick, Coventry CV4 7AL, U.K.

## Abstract

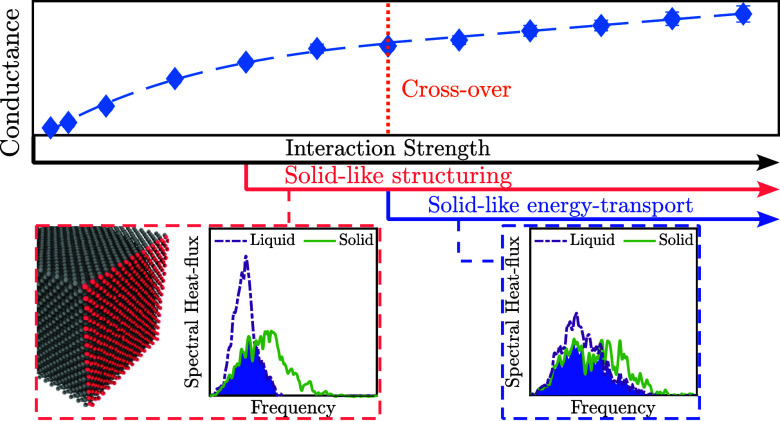

The interfacial thermal conductance at a solid/liquid interface (*G*) exhibits an exponential-to-linear crossover with increasing solid/liquid interaction strength, previously attributed to the relative strength of solid/liquid to liquid/liquid interactions. Instead, using a simple Lennard-Jones setup, our molecular simulations reveal that this crossover occurs due to the onset of solidification in the interfacial liquid at high solid/liquid interaction strengths. This solidification subsequently influences interfacial energy transport, leading to the crossover in *G*. We use the overlap between the spectrally decomposed heat fluxes of the interfacial solid and liquid to pinpoint when “solid-like energy transport” within the interfacial liquid emerges. We also propose a novel decomposition of *G* into (i) the conductance right at the solid/liquid interface and (ii) the conductance of the nanoscale interfacial liquid region. We demonstrate that the rise of solid-like energy transport within the interfacial liquid influences the relative magnitude of these conductances, which in turn dictates when the crossover occurs. Our results can aid engineers in optimizing *G* at realistic interfaces, critical to designing effective cooling solutions for electronics among other applications.

## Introduction

Rising transistor densities and on-chip clock speeds have yielded increasing thermal loads on modern integrated circuits (ICs), leading to a severe degradation in their performance.^1,2^ As IC cooling is primarily limited by modern single-phase thermal management technologies,^3^ this has spurred interest in two-phase cooling devices, which exploit the latent heat of vaporization of the working fluid to extract a significantly greater amount of heat from ICs.^4−6^ Traditionally, the interfacial thermal conductance across the solid/liquid interface (*G*) has been disregarded in such processes, given the greater importance of conduction, evaporation, and diffusion in the bulk liquid. However, in emerging micro- and nanoengineered systems, *G* is much more likely to be the bottleneck to performance improvements compared to bulk processes.^7^ Despite the growing relevance of *G* in the thermal management of ICs, as well as its influence on the heat transfer across plasmonic nanoparticles in their various applications,^8−10^ such as drug delivery,^11−14^ medical imaging,^15,16^ and thermal therapies,^17−22^ the nanoscale origins of *G* remain poorly understood.^23^

Early experimental studies carried out with the goal of optimizing *G* showed that it is strongly influenced by the surface wettability. In these investigations, the wettability was controlled by coating the solid with self-assembled monolayers (SAMs) comprising different functional groups, thus tuning the strength of solid/liquid interfacial interactions.^24^ Ge et al.^25^ and Tian et al.^26^ showed that coating aluminum/water, gold/water, and gold/ethanol interfaces with philic SAMs can enhance *G* by a factor of 2–5 compared to phobic SAMs. Harikrishna et al.^27^ generalized this trend for functionalized gold/water interfaces by reporting a linear correlation between *G* and the work of adhesion *W*_SL_, where *W*_SL_ is an energetic quantification of surface wettability.

Subsequent experimental investigations have contested the existence of such a linear correlation between *G* and wettability. Park and Cahill^28^ evaluated *G* at SAM-coated gold nanodisks suspended in water–ethanol and water–surfactant mixtures of various concentrations, reporting a nonlinear relation between *G* and *W*_SL_ for both hydrophilic and hydrophobic SAMs. Tomko et al.^29^ explored gold/water, gold/hydrocarbon, and gold/fluorocarbon interfaces, finding *G* to be unrelated to *W*_SL_ across these interfaces. Similar discrepancies have been observed in the closely related field of heat transfer across solid/solid interfaces.^30−33^ Coupled with the challenges of conducting experimental studies,^34^ these hinder our ability to understand how wettability influences *G*. Consequently, the use of numerical modeling has gained significance.

Molecular dynamics (MD) simulations have been widely deployed, with a number of studies finding *G* to be linearly correlated to the surface wettability. In alignment with experimental practices, some studies controlled the wettability through the implementation of SAMs, reporting a linear relationship between *G* and *W*_SL_.^35−37^ However, a large number of MD studies investigating *G* opted to directly alter the solid/liquid interaction strength ε_SL_, rather than manipulating interfacial interactions via the SAM functional group.^38−47^ Through this artificial “wettability-tuning” approach, Alexeev et al.^48^ found *G* to be linearly correlated to *W*_SL_ at graphene/water interfaces. Similarly, Ramos-Alvarado et al.^49^ noted a linear relation between *G* and *W*_SL_ at silicon/water interfaces for both crystallographic planes studied. These MD studies, regardless of whether the wettability was varied using SAMs or artificially tuned, consistently reproduce the linear relationship with *G* reported experimentally.^27^

However, in other MD investigations, the relationship between wettability and *G* was found to be nonlinear across the range of wettabilities considered. Wei et al.^50^ noted that the relation between *G* and *W*_SL_ at SAM-coated gold/organic-liquid interfaces could not be accurately described by a simple linear fit. Qian et al.^42^ investigated *G* at a graphene/ionic-liquid interface, varying the surface wettability by tuning ε_SL_ and subsequently computing the resulting magnitude of *W*_SL_. They reported a complex correlation between *G* and *W*_SL_, characterized by a steep gradient at low wettabilities, followed by a reduction in the slope beyond a certain threshold. Xue et al.^51^ studied the impact of artificially tuning wettability at a simple Lennard-Jones (LJ) solid/liquid interface, directly relating *G* to the wetting parameter ε_SL_, rather than the *W*_SL_ values produced by altering ε_SL_. They discovered a “crossover” from an exponential regime to a linear one with a reduction in the slope at the crossover threshold, hypothesizing that this occurs once ε_SL_ exceeds the liquid/liquid interaction strength ε_LL_, which signifies the transition from a “nonwetting” to a “wetting” interface. However, this explanation is not universal, as evidenced by other studies where the shift from nonwetting to wetting interfaces did not produce a crossover in *G*.^35,36^ Therefore, the mechanisms governing the nature of the relationship between *G* and solid/liquid interaction strength remain unclear.

Efforts have been dedicated to comprehending the relationship between *G* and wettability through the examination of the *structural properties* of the interfacial liquid. Alexeev et al.^48^ demonstrated a link between peak interfacial liquid density and *G* at a graphene/water interface; however, other studies have shown that this cannot be used as the sole predictor of *G*.^39,52−58^ Ma et al.^39^ showed that the enhancement in *G* resulting from the deposition of patterned surface charges at a graphene/water interface could be attributed to an increase in interfacial liquid in-plane ordering, but this has since been shown to not be universal.^52,53,59^ Ramos-Alvarado et al.^49^ identified a correlation between the density depletion length and *G* at silicon/water interfaces for multiple crystallographic planes of silicon, but this could not be extended to other interfaces.^45,53,57,60,61^ Note that as these solid/liquid systems are electrically nonconductive, atomic vibrations are the sole mechanism of energy transport.^23^ While structural variations in the interfacial region have been observed, it is not clear how these relate to the vibrational coupling between the two phases.

An alternative approach to gain insights into the relationship between *G* and ε_SL_ is to analyze atomic vibrations directly by using *spectral techniques*. Qian et al.^62^ demonstrated that the enhancement in *G* yielded by increasing the surface charge magnitude at graphene/ionic-liquid interfaces is accompanied by a rise in the coupling of high-frequency vibrations between the interfacial solid and liquid. Alosious et al.^63^ illustrated that the rise in *G* due to the reduction in confinement at a carbon-nanotube/water interface is associated with an increase in the similarity of vibrations between the solid and liquid. Zhou et al.^64^ showed that the enhancement in *G* due to the presence of atomic defects at graphene/hydrocarbon interfaces is linked to an increase in the coupling of in-plane vibrations between the interfacial solid and liquid. Importantly, Sääskilahti et al.^65^ demonstrated that the rise in *G* resulting from an increase in artificially tuned wettability at an LJ solid/liquid interface is associated with an enhancement in the coupling of high-frequency vibrations from the solid to the liquid, as well as an improvement in the coupling of in-plane vibrations. Gonzalez-Valle and Ramos-Alvarado^45^ observed a similar effect when varying ε_SL_ at silicon-carbide/water interfaces. Furthermore, Giri et al.^66^ noted that the exponential-to-linear regime crossover observed by Xue et al.^51^ is associated with enhanced coupling of in-plane vibrations between the interfacial solid and liquid around the crossover threshold. These studies underscore that directly investigating interfacial heat carriers can provide insights into the nonlinearity of the relationship between *G* and wettability.

In this work, we uncover the structural and spectral mechanisms driving the widely reported exponential-to-linear crossover in *G* with increasing ε_SL_.^38,42,51,66,67^ We use a simple LJ solid/liquid interface as this enables us to isolate the effects of ε_SL_ alone on *G*. We develop a new spectral metric that better captures the underpinning physics of the regime crossover. Our results have broad significance for interfacial heat transfer, and our metric can be readily extended to more complex interfaces in future work.

## Methodology

### System Setup

**Figure 1:**
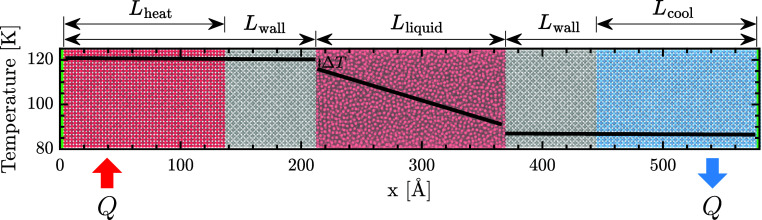
Typical temperature distribution yielded by the steady-state NEMD simulation superposed on a schematic of the system studied. The green-colored solid layers at both ends of the domain are held rigid to avoid the displacement of the interface. In the left wall, heat *Q* = 80 meV/ps is injected into the red-colored portion of the wall of length *L*_heat_. In the right wall, *Q* is extracted from the blue-colored portion of the wall of length *L*_cool_. The gray-colored portions of both walls are permitted to vibrate freely. A line of best fit is computed through the temperature distribution of the bulk liquid and is then extrapolated to the interfacial solid. The temperature discontinuity at the interface (Δ*T*) is then determined and used to compute the interfacial thermal conductance.

We conduct nonequilibrium MD (NEMD) simulations using LAMMPS^68^ to investigate the ε_SL_-driven regime crossover in *G* at an LJ solid/liquid interface. We consider a liquid confined between the walls of a nanochannel separated by a distance *L*_liquid_ = 155 Å (see Figure 1). The solid layers at both ends of the domain, colored green, are held rigid to avoid displacement of the interface. In the left wall, adjacent to the green atoms lies a portion of red-colored solid of extent *L*_heat_ where heat *Q* = 80 meV/ps is injected. Similarly, in the right wall lies a blue-colored portion of solid of extent *L*_cool_ where heat *Q* is extracted. The gray portions of both walls are allowed to vibrate freely. From the generated temperature distribution, the interfacial temperature discontinuity Δ*T* can be obtained by extrapolating the line of best fit through the bulk liquid to the interfacial solid, as overlaid in Figure 1. The interfacial thermal conductance is then determined using *G* = *Q*/*A*Δ*T*, where *A* is the cross-sectional area. All interactions are governed by the LJ potential^69^ with a cutoff distance *r*_cut_ = 8.5 Å. Additional simulation details are given in Section S1 of the Supporting Information.

### Choice of ε_SL_ Range

The solid/liquid interaction strength ε_SL_ is systematically varied from 2.5 to 100 meV to capture the crossover in *G* observed in previous studies.^38,51,66,67^ The surface wettability is quantified by using the cylindrical droplet method to calculate the contact angle (θ) for the range of values chosen for ε_SL_, as detailed in Section S2 of the Supporting Information. The resulting values of θ are presented in Table 1, where we observe full wetting of the interface beyond ε_SL_ = ε_LL_ = 10.3 meV. Note that this means that, for a majority of the ε_SL_ values studied here, the surface is fully wetting, in line with prior studies.^38,40,51,66,67^

**Table 1:** Values of Contact Angle (θ) Resulting from Various Magnitudes of the Solid/Liquid Interaction Strength (ε_SL_)

ε_SL_ [meV]	θ [°]
2.5	172.6
4	126.7
5	100.8
6	76.8
7.5	34.5
10.3	0

## Results and Discussion

### Crossover Threshold

**Figure 2:**
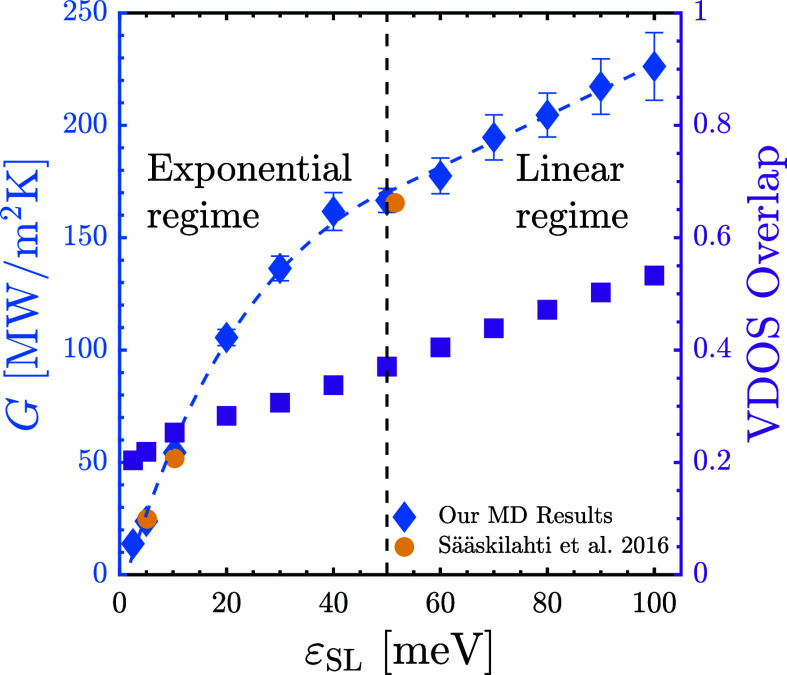
Interfacial thermal conductance (*G*) and interfacial VDOS Overlap plotted against the solid/liquid interaction strength (ε_SL_). MD results generated by this study are validated against results by Sääskilahti et al.^65^ The dashed line indicates the crossover in *G* vs ε_SL_ from an exponential regime to a linear regime. No crossover is seen in VDOS Overlap vs ε_SL_.

In Figure 2, we plot the variation of *G* with ε_SL_. As previously shown, *G* increases exponentially at low values of ε_SL_, followed by a linear dependence for high values of ε_SL_.^38,51,66,67^ Our results agree well with those of Sääskilahti et al.,^65^ who studied a similar system. However, their study included a limited set of ε_SL_ values, thus failing to observe the crossover (see Section S3 of Supporting Information for additional comparisons). By investigating *G* for a broader range of ε_SL_ values, we clearly observe the crossover to occur around ε_SL_ ≈ 50 meV, at which point the liquid fully wets the solid.

### Structural Investigation of Crossover

To assess the influence of the out-of-plane (i.e., perpendicular to the interface) structural features of the interfacial liquid on the crossover, the interfacial density layering is computed. For ε_SL_ > 30 meV, the first liquid layer fully adheres, while the second layer does not adhere even at ε_SL_ = 100 meV (see Section S4 of Supporting Information). Similarly, the impact of the in-plane (i.e., parallel to the interface) structural features of the interfacial liquid on the crossover is studied by calculating the radial distribution functions (RDFs) of its individual layers. The first two layers of the interfacial liquid exhibit long-range in-plane ordering for ε_SL_ > 30 meV (see Section S5 of Supporting Information). The pronounced out-of-plane layering observed in the interfacial density fluctuations and the significant degree of in-plane ordering present in the RDF calculations can both be attributed to the range of values chosen for ε_SL_, most of which correspond to a fully wetted surface. We denote these ε_SL_-induced structural changes in the interfacial liquid as “solid-like structuring” (SS). While interfacial liquid SS has previously been observed in complex systems both experimentally and numerically,^70−74^ it was induced by electrostatic interfacial forces and hydrogen bonding. In contrast, the SS observed in the simple LJ system here results from the extremely high values of ε_SL_. Nonetheless, the onset of SS at ε_SL_ ≈ 30 meV, while potentially relevant to the mechanism driving the crossover in *G*, precedes the crossover threshold of ε_SL_ ≈ 50 meV.

### Spectral Investigation of Crossover

#### Vibrational Density of State Overlap

A prominent spectral metric to analyze *G* is the vibrational density of states (VDOS), which quantifies the vibrational modes existing within a specific frequency range,^75^ and is used to identify the permissible vibrational frequencies in a medium. The VDOS of the interfacial solid (VDOS_IS_) is computed using the Fourier transform of the velocity autocorrelation function of the atoms contained in the outermost layer of the solid, and the VDOS of the interfacial liquid (VDOS_IL_) is similarly computed using the atoms within the first adhered liquid layer^76^ (see Section S7 of Supporting Information). *G* has been related to the degree of similarity in the vibrations of the first solid and liquid layers, which is quantified by the area overlap between the VDOS_IS_ and VDOS_IL_ distributions.^45,54,77−88^ This “VDOS Overlap” metric is computed for all values of ε_SL_ considered and plotted in Figure 2. We find that the VDOS Overlap increases linearly with ε_SL_, presenting no distinctive features around the crossover threshold. Hence, like SS, the VDOS Overlap metric cannot explain the crossover.

The spectral decomposition of heat flux (SDHF) is an alternative spectral metric that quantifies the individual contribution of each mode to the total energy transported in a medium, rather than merely quantifying the permissible vibrational frequencies. It is evaluated via the inner product of the Fourier transforms of the cumulative force experienced by all atoms involved in energy transport (i.e., atoms within the interaction cutoff distance *r*_cut_)^65^ and their velocities (see Section S6 of Supporting Information). The SDHF of the interfacial solid (SDHF_IS_) thus represents the modes engaged in *transmitting* thermal energy to the liquid,^65^ while the SDHF of the interfacial liquid (SDHF_IL_) represents the modes engaged in *receiving* thermal energy from the solid.^65,89^

While SDHF_IS_ has been studied extensively,^39,45,58,64,88,90−92^ the dependence of SDHF_IL_ on ε_SL_ is systematically studied for the first time here. By computing SDHF_IS_ and SDHF_IL_ for all values of ε_SL_ and contrasting these with VDOS_IS_ and VDOS_IL_, respectively, we are also able to identify why the VDOS Overlap fails to explain the crossover.

#### Contrasting VDOS_IS_ and SDHF_IS_

**Figure 3:**
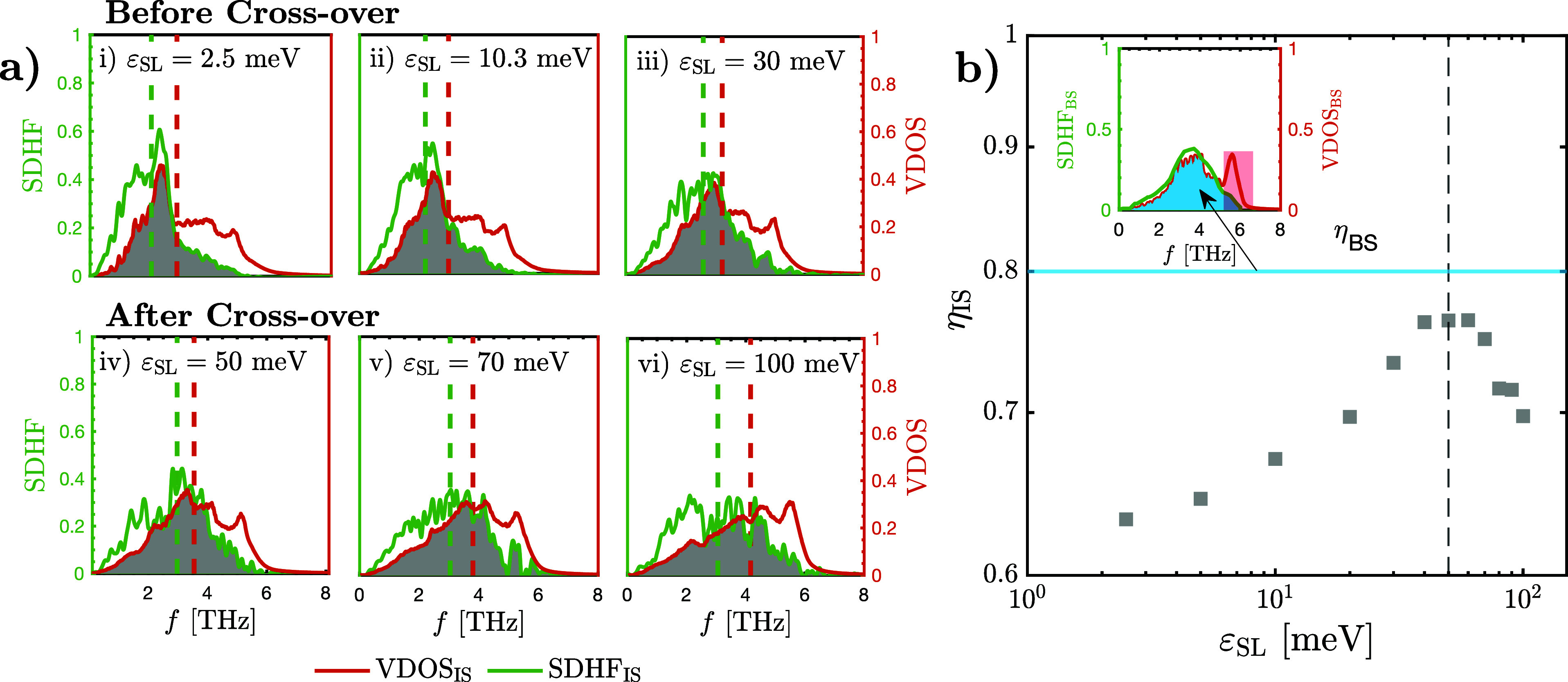
(a) Normalized VDOS_IS_ and SDHF_IS_ for different values of solid/liquid interaction strength (ε_SL_). The top three subplots depict the behavior of the spectra prior to the crossover, while the bottom three depict the spectra after it. The dashed orange and green lines represent the median lines of VDOS_IS_ and SDHF_IS_, respectively. The gray shaded regions denote the area overlap between VDOS_IS_ and SDHF_IS_, which is denoted as η_IS_. (b) Variation of η_IS_ with ε_SL_. The dashed line represents the crossover in *G*, which coincides with the onset of the plateau in η_IS_. The inset illustrates the normalized VDOS and SDHF in the bulk of the solid. The area overlap of the bulk solid VDOS and SDHF is denoted as η_BS_ (solid blue line), which acts as an upper limit for the area overlap between the solid’s VDOS and its SDHF. The red shaded area in the inset highlights the discrepancy between the VDOS and SDHF at high frequencies in the bulk.

Figure 3a illustrates the normalized distributions of SDHF_IS_ and VDOS_IS_ for different values of ε_SL_ before [Figure 3a(i–iii)] and after the crossover [Figure 3a(iv–vi)]. From these plots, we make four observations:

1. At the lowest value of ε_SL_, VDOS_IS_ possesses a distinct low-frequency peak (near 2 THz), as seen in Figure 3a(i). With increasing ε_SL_, this low-frequency peak becomes gradually suppressed, while enhancements at the middle (2–5 THz) and high (>5 THz) frequencies become more prominent [see Figure 3a(ii–vi)]. This has been shown to result due to the increased adhesion of the interfacial liquid.^40,66^ To gauge the effect of these changes on the overall distribution of VDOS_IS_, we have added dashed red lines corresponding to the median value of each VDOS_IS_ distribution. VDOS_IS_ exhibits a rightward shift when ε_SL_ is increased, as indicated by the movement of its median line to the right. This signifies the increased *availability* of high-frequency modes.
2. Analogously, SDHF_IS_ is also characterized by a pronounced low-frequency peak near 2 THz at the lowest value of ε_SL_ [Figure 3a(i)]. It similarly experiences low-frequency damping and enhancements at the middle and high frequencies with rising ε_SL_. As with VDOS_IS_, we quantify the effect of these changes using dashed green lines, corresponding to the median value of each SDHF_IS_ distribution. The rightward movement of this median line with increasing ε_SL_ demonstrates a right-shifting in SDHF_IS_ [see Figure 3a(ii–vi)]. As previously established, this occurs due to the increase in the *utilization* of middle and high-frequency modes in transferring heat to the liquid.^45,65^
3. Analyzing the relative positions of the median lines of the VDOS_IS_ and SDHF_IS_ distributions, we show—for the first time—that SDHF_IS_ exhibits more pronounced right-shifting and mid-to-high-frequency enhancements compared to VDOS_IS_ until the crossover. This is quantified by the diminishing gap between the red and green dashed median lines with increasing ε_SL_ in Figure 3a(i–iv). At the lowest value of ε_SL_, as SDHF_IS_ is broadly to the left of VDOS_IS_, the relatively larger right-shifting in SDHF_IS_ results in an increasing overlap between the SDHF_IS_ and VDOS_IS_ distributions with increasing ε_SL_, until the crossover threshold [Figure 3a(i–iv)]. This suggests a greater increase in the utilization of mid-to-high-frequency modes compared to the increase in their availability. Beyond the crossover, the relative rate of right-shifting in SDHF_IS_ drops off, while VDOS_IS_ continues its monotonic right-shifting, leading to a divergence of the two distributions [Figure 3a(iv–vi)]. This is quantified by the widening gap between the median lines and suggests that the rise in the availability of middle- and high-frequency modes is greater than the rise in their utilization after the crossover.
4. Overall, there is a notable discrepancy between the SDHF_IS_ and VDOS_IS_ distributions at high frequencies for all values of ε_SL_, which will be elaborated upon later.

Analogous to the VDOS Overlap in Figure 2, we quantify the similarity between the VDOS_IS_ and SDHF_IS_ distributions using their area overlap (η_IS_), plotted in Figure 3b. Note that unlike the VDOS Overlap, which attempts to quantify interfacial energy transport, η_IS_ is purely a solid-side measurement calculation and captures the utilization of modes by the solid for energy transport relative to their availability. As seen in Figure 3b, η_IS_ rises to a maximum at the crossover threshold (≈75%), followed by a plateau and a subsequent decline. Even at its maximum, η_IS_ is significantly below 100%, confirming that the discrepancy between the VDOS_IS_ and SDHF_IS_ distributions at high frequencies in Figure 3a causes a quantifiable disparity between the two spectra across all values of ε_SL_. For reference, we also compute the area overlap between the SDHF and VDOS distributions of the bulk solid (η_BS_, which is independent of ε_SL_), as shown in the inset of Figure 3b. There is a similar discrepancy between the two distributions at high frequencies (shaded red), demonstrating that the identified limitations of VDOS-based analyses persist across the entire solid, despite the higher efficiency of heat transfer within the bulk.

#### Comparing VDOS_IL_ and SDHF_IL_

**Figure 4:**
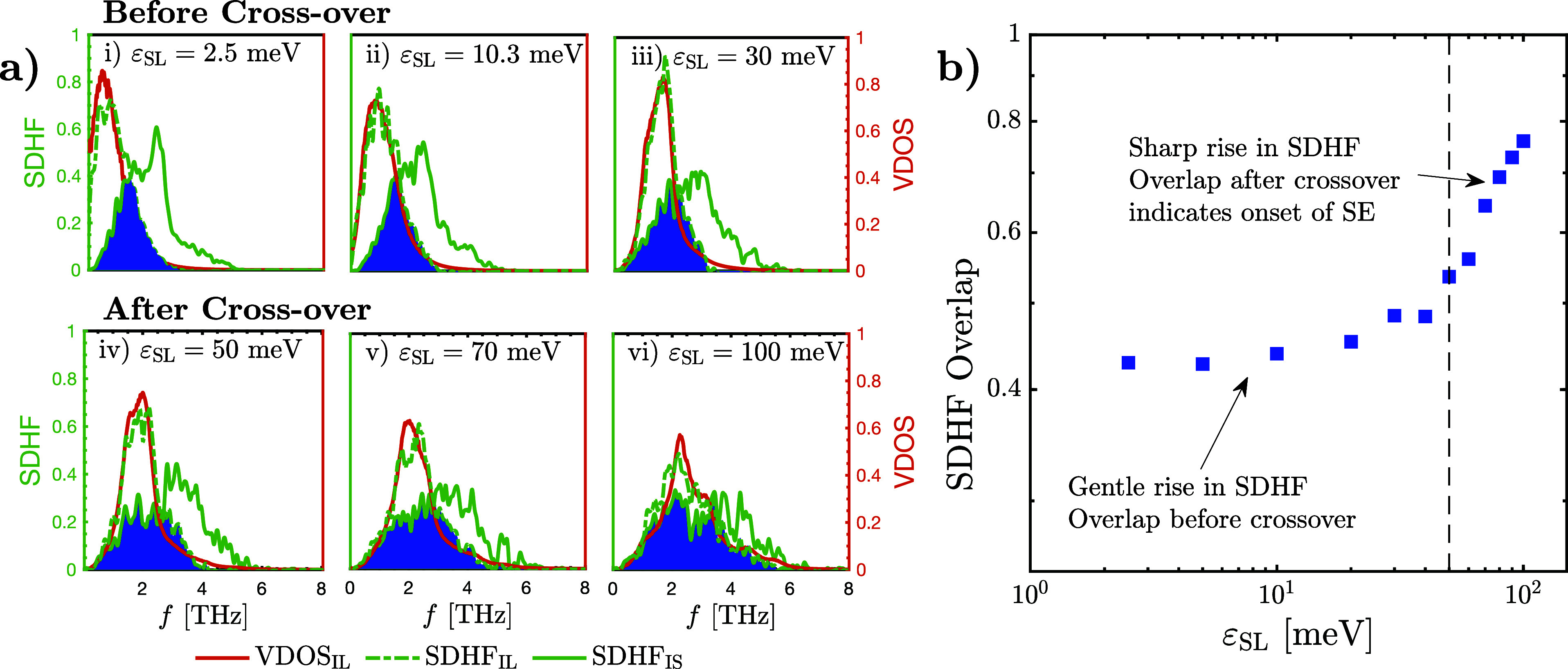
(a) Normalized VDOS_IL_ as well as the SDHF_IL_ and SDHF_IS_ for select values of the solid/liquid interaction strength ε_SL_. The blue shaded regions denote the area overlap between SDHF_IS_ and SDHF_IL_, referred to as the SDHF Overlap. (b) SDHF Overlap plotted against ε_SL_. The dashed line represents the crossover threshold from the first regime of *G* into the second, which occurs close to where a distinct change in the slope of SDHF Overlap vs ε_SL_ is observed.

The normalized VDOS_IL_, SDHF_IL_, and SDHF_IS_ distributions are presented in Figure 4a for specific values of ε_SL_. Here, we show for the first time that the VDOS_IL_ and SDHF_IL_ distributions are nearly identical for all values of ε_SL_, making their area overlap near-perfect (η_IL_ ≃ 1). This implies that the VDOS serves as an accurate representation of the modes involved in thermal transport *only within the liquid*. Therefore, the inability of the VDOS Overlap metric to explain the regime crossover is likely because VDOS_IS_ fails to accurately depict thermal transport in the solid, as established in Figure 3 by the discrepancies between the VDOS_IS_ and SDHF_IS_ distributions.

#### Comparing SDHF_IS_ and SDHF_IL_

At the lowest value of ε_SL_ in Figure 4a(i), we can see that SDHF_IL_ possesses a narrow distribution with a low-frequency peak (near 1 THz). Here, the SDHF_IL_ distribution is vastly dissimilar to that of SDHF_IS_, with the breadth of the SDHF_IS_ distribution significantly exceeding that of SDHF_IL_. This demonstrates that, at this magnitude of ε_SL_, the solid transmits a significant amount of energy beyond the maximum permissible vibrational frequency in the liquid. Increasing ε_SL_ to 30 meV, SDHF_IL_ is seen to right-shift substantially, now being characterized by a peak at ≈2 THz, as seen in Figure 4a(i–iii). Despite this, SDHF_IL_ remains distinctly dissimilar to SDHF_IS_ below the crossover threshold, maintaining a considerably narrower distribution. This finding confirms the hypothesis proposed by Sääskilahti et al.^65^ that high-frequency modes in the solid are redistributed into lower-frequency modes within the liquid due to the inherent limitations on the existence of such high-frequency modes in the liquid.

However, beyond the crossover threshold, we make an interesting observation: SDHF_IL_ begins broadening, as shown in Figure 4a(iv–vi). Its peak incurs a reduction in magnitude, and the maximum permissible vibrational frequency in the liquid rises to match the maximum permissible frequency in the solid at ε_SL_ = 100 meV. Thus, the SDHF_IL_ distribution begins resembling that of SDHF_IS_ to an increasing degree beyond the crossover. At the highest values of ε_SL_, the hypothesis by Sääskilahti et al.^65^ no longer holds, as the liquid significantly engages the high-frequency modes seen in the solid in heat transfer. Qualitatively, this indicates the onset of “solid-like energy transport” (SE) beyond the crossover.

By computing the area overlap between the SDHF_IS_ and SDHF_IL_ distributions (SDHF Overlap), we can accurately track the relation between ε_SL_ and interfacial energy transport, avoiding any overestimation of high-frequency mode contributions associated with the VDOS Overlap. This new SDHF Overlap metric is computed for all values of ε_SL_ and is shown in Figure 4b. It can be seen that the SDHF Overlap has a weak dependence on ε_SL_ when ε_SL_ < 50 meV. However, a distinct change in the slope occurs around the crossover threshold. This indicates the start of SE in the interfacial liquid, the threshold for which differs from that of the interfacial liquid SS identified by the density layering and RDF calculations (ε_SL_ ≈ 30 meV). The SS in the interfacial liquid indicates that its structure begins resembling that of a solid; however, the interfacial liquid SE threshold identifies when it starts engaging its modes in a solid-like manner.

### Connecting the Structural and Spectral Descriptions of Crossover

**Figure 5:**
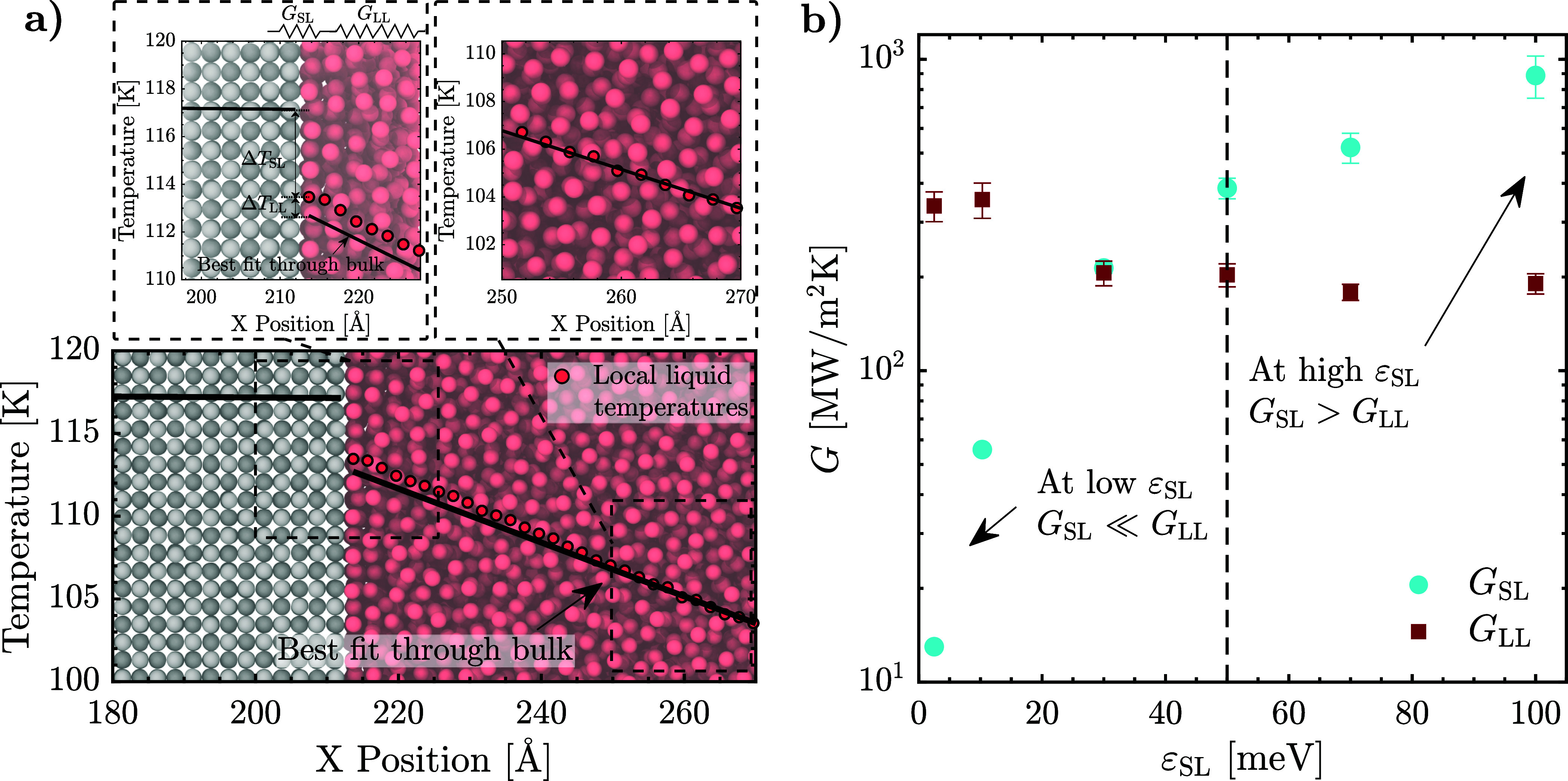
(a) Simulation snapshot demonstrating the deviation of local interfacial liquid temperatures (red points) from the line of best fit through the bulk (black line). The left inset illustrates our proposed decomposition of the temperature discontinuity into two components: the localized temperature discontinuity between the solid and the first adhered liquid layer (Δ*T*_SL_) and the temperature difference between the actual temperature of the adhered liquid layer and the extrapolated intercept at the same location of the line fitted to the temperature field in the bulk liquid (Δ*T*_LL_). The right inset shows a portion of the fit through the temperatures of the bulk liquid. (b) Two components of *G* plotted against the solid/liquid interaction strength (ε_SL_). *G*_SL_ is computed from Δ*T*_SL_ and represents the localized thermal conductance between the outermost solid layer and the adjacent liquid layer. *G*_LL_ is computed from Δ*T*_LL_ and quantifies the effective thermal conductance of the remainder of the layered liquid. The dashed line represents the crossover threshold from the exponential regime of *G* into the linear regime, which occurs where *G*_SL_ first exceeds *G*_LL_, associated with the onset of SE in the interfacial liquid.

To better understand how the interfacial liquid SS and SE influence the crossover, we return to the definition of *G*. The results presented in Figure 2 are obtained via the conventional approach, where the total temperature discontinuity Δ*T* is calculated by extrapolating the line of best fit through the temperature profile of the bulk liquid to the interfacial solid.^93^ However, considering the changes in the structural characteristics and energy-transport properties of the interfacial liquid, we closely examine the localized temperatures of the interfacial liquid layers. We show that Δ*T*, in fact, comprises two components (see the left inset in Figure 5a): (i) the difference between the local temperatures of the interfacial solid layer and the first interfacial liquid layer, denoted by Δ*T*_SL_ and (ii) the difference between the local temperature of the first interfacial liquid layer and the extrapolated value from the best linear fit to the bulk temperature field, denoted by Δ*T*_LL_. As evidenced by the insets of Figure 5a, the local temperatures in the liquid deviate from the linear fit significantly in the density layering region (see Section S4 of Supporting Information). Δ*T*_LL_ has not been quantified prior to this study; its existence reveals the fact that the layered liquid has a lower thermal conductivity than the bulk.

Using Δ*T*_SL_ and Δ*T*_LL_, we can effectively decompose *G* into two thermal conductances, *G*_SL_ = *Q*/*A*Δ*T*_SL_ and *G*_LL_ = *Q*/*A*Δ*T*_LL_, respectively (see the left inset of Figure 5a). *G*_SL_ represents the localized thermal conductance between the outermost solid layer and the adjacent liquid layer, while *G*_LL_ quantifies the effective thermal conductance of the remainder of the layered liquid. Figure 5b shows the variation of *G*_SL_ and *G*_LL_ with ε_SL_. For the lowest values of ε_SL_, *G*_SL_ is significantly smaller than *G*_LL_, making *G*_SL_ the bottleneck in the conductance network. With rising ε_SL_, *G*_SL_ increases, while *G*_LL_ decreases sharply around ε_SL_ = 30 meV then remains nearly unchanged. This occurs around the onset of SS in the interfacial liquid and corresponds to the reduction in conductance due to the absence of diffusion in the adhered liquid. As *G*_SL_ is the limiting factor at low values of ε_SL_, its increase has a significant impact on the total *G* before crossover. As such, the initial steep gradient in total *G* seen in Figure 2 prior to the crossover stems from the large increase in *G*_SL_ relative to *G*_LL_.

*G*_SL_ eventually rises significantly above *G*_LL_ around ε_SL_ = 50 meV, marked by the onset of SE. Near this threshold, the temperature of the adhered liquid practically becomes an extension of the temperature profile of the solid, causing Δ*T*_SL_ to drop below Δ*T*_LL_. Beyond this point, *G*_LL_ becomes the bottleneck in the conductance network. With rising ε_SL_, further increases in *G*_SL_ thus no longer influence the total *G* to the same extent, explaining the reduction in the slope in *G* vs ε_SL_ incurred beyond the crossover in Figure 2. The crossover can thus be understood as the point separating regimes where either *G*_SL_ or *G*_LL_ is the primary limiting factor in the overall conductance network. While the sharp reduction in *G*_LL_ brought about by the interfacial liquid SS plays a role in causing *G*_SL_ to be above *G*_LL_, it is only upon the onset of SE where *G*_SL_ becomes notably higher than *G*_LL_. As such, the shift in bottleneck from *G*_SL_ to *G*_LL_ results due to a combination of the interfacial liquid SS and SE.

We therefore show for the first time that the exponential regime, referred to as the nonwetting regime in the literature, actually manifests when *G*_SL_ ≪ *G*_LL_. Similarly, the linear regime, referred to as the wetting regime in the literature, in turn manifests when *G*_SL_ > *G*_LL_. The transition between the two regimes is not driven by the relative importance of solid/liquid to liquid/liquid interactions as previously suggested^51,66^ but instead is driven by the interfacial liquid SS and SE, as indicated by our new SDHF Overlap metric in Figure 4b.

## Conclusions

In this work, we investigate the poorly understood nonlinearity between *G* and ε_SL_ that arises at certain solid/liquid interfaces. To achieve this, we employ nonequilibrium MD simulations to elucidate the ε_SL_-driven regime crossover at an LJ solid/liquid interface,^51,66^ examining both the *structural* and *spectral* properties of the interface at several values of ε_SL_ before and after the crossover.

We find that the crossover cannot be fully attributed to the in-plane and out-of-plane structure of the interfacial liquid and that the conventional VDOS Overlap spectral metric similarly fails to explain it. Instead, by focusing on the actual modes involved in heat transfer through studying the SDHF on both sides of the interface, we are able to explain why this crossover occurs. Our SDHF Overlap metric indicates that the crossover is partly characterized by the onset of SE in the interfacial liquid. We also propose a novel decomposition of Δ*T* into Δ*T*_SL_ and Δ*T*_LL_ and use them to compute interfacial thermal conductances *G*_SL_ and *G*_LL_, respectively. The relative magnitude of these two conductances is found to be associated with the regime crossover, rather than the relative strength of the interfacial solid/liquid to liquid/liquid interactions. In future work, our approach can be extended to more realistic solid/liquid interfaces in order to further our understanding of *G*.

## Supporting Information Available

Numerical methodology and validations, as referenced in the main text (PDF)

## Notes

The data necessary to reproduce the figures in the publication, as well as the post-processing scripts used to generate this data, are openly available in the Edinburgh DataShare repository at https://doi.org/10.7488/ds/7725
