# Supplementary material for: Unraveling the Regimes of Interfacial Thermal Conductance at a Solid/Liquid Interface
Source: J Phys Chem C Nanomater Interfaces. 2024 May 13;128(20):8408–17. doi: 10.1021/acs.jpcc.4c00536 (PMC11129300; doi:10.1021/acs.jpcc.4c00536)
Supplement: Supplementary file 1 — jp4c00536_si_001.pdf [file jp4c00536_si_001.pdf]

Unraveling the Regimes of Interfacial Thermal Conductance at a Solid/Liquid Interface: Supporting Information

Abdullah El-Rifai[1], Sreehari Perumanath[2], Matthew K. Borg[1], and Rohit Pillai[1]

[1]Institute for Multiscale Thermofluids, University of Edinburgh, Edinburgh EH9 3FD, United Kingdom
[2]Mathematics Institute, University of Warwick, Coventry CV4 7AL, United Kingdom

April 8, 2024

1 Molecular Dynamics

1.1 Additional Simulation Details

The energy scale in the liquid-liquid and solid-solid LJ interaction potentials are kept at $\varepsilon_{LL} = 10.3$ meV and $\varepsilon_{SS} = 10\varepsilon_{LL}$, respectively.[1–4] For all interactions, the length scale in the LJ potential is kept at $\sigma = 3.4$ Å. All atomic masses m are set to 39.948 amu.

The domain boundaries are periodic except along the x direction, for which we used the 'fixed' boundary condition in LAMMPS. The system is initally equilibrated at 100 K and 50 MPa in a micro-canonical ensemble for 2.5 ns. The pressure is controlled by iteratively adjusting the number of liquid atoms such that the density in the bulk of the liquid is the same (≈ 1370 kg/m^3) across all values of ε_{SL}. Following equilibration, heat Q is continuously injected and extracted from the left and right walls as detailed in the main article for a duration of 10 ns.

1.2 Finite Size Effects Study

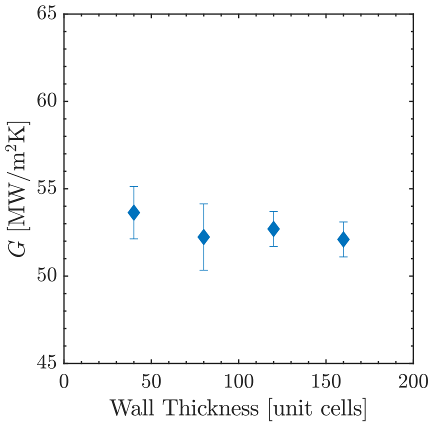

Figure S1: Interfacial thermal conductance (G) computed for the solid/liquid interaction strength ε_{SL} = 10.3 meV at various wall thicknesses. No dependence on wall thickness is found beyond 40 unit cells, indicating the absence of finite size effects.

We model our system (Figure 1 of the main article) based on that of Sääskilahti et al.,[1] with adjustments to ensure that there are no finite-size effects. Sääskilahti et al.[1] demonstrated that

interfacial thermal conductance (G) is not impacted by the fluid channel length (L_{liquid} = 55 Å). However, we choose to increase L_{liquid} to 155 Å so as to ensure confinement effects are avoided, as we test for higher wettabilities. The total thickness of the solid walls L_{wall} is 40 unit cells, with $L_{heat} = L_{cool} = 25$ unit cells thick. The unit cell constant is 1.56σ. The walls comprise a face-centred cubic (FCC) crystal with its [100] crystallographic plane oriented in the x-direction. A finite-size effects study was conducted to confirm that G is independent of wall thickness beyond 40 unit cells, as seen in Figure S1, for ε_{SL} = 10.3 meV. Sääskilahti et al.[1] found that G is independent of cross-sectional dimensions beyond 8×8 unit cells. Nonetheless, we model a larger cross-sectional area of 15×15 unit cells to improve the statistical accuracy of our results.

2 Contact Angle Simulations

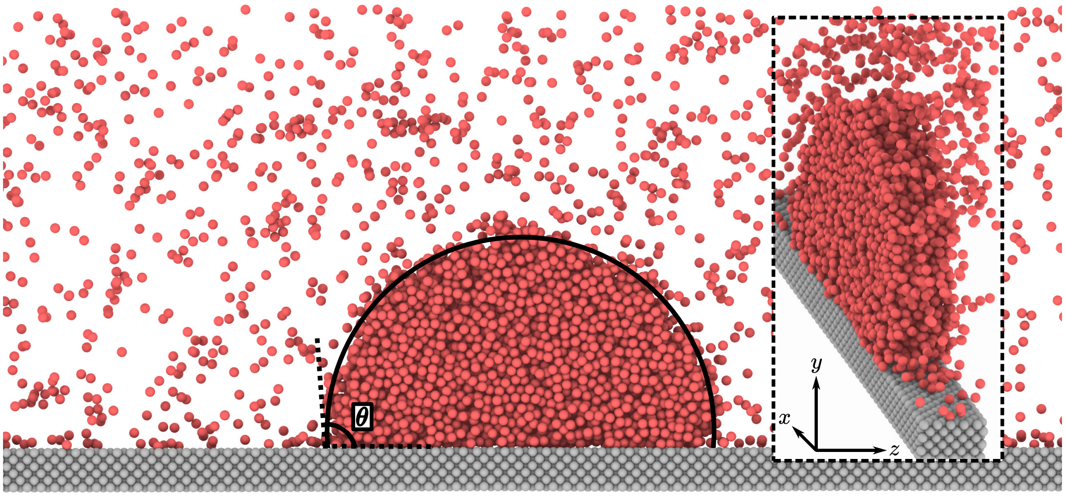

Figure S2: An illustration of the method employed to compute the contact angle (θ) of quasi-2D cylindrical droplets. The inset depicts a three-dimensional view of the droplet.

To quantify the relationship between ε_{SL} and wettability when the surface is partially wetted, the contact angles (θ) of quasi-2D cylindrical droplets are calculated at various magnitudes of ε_{SL}. A portion of the simulation domain implemented to achieve this is shown in Figure S2, where the inset depicts a three-dimensional view of the droplet in the x,y, and z directions. The domain boundaries are periodic except along the y direction, for which we used the 'fixed' boundary condition in LAMMPS. This prevents condensation along the periodic image of the wall that would result at top of the domain. A fictitious reflective wall is placed at the top of the domain using the 'fix wall/reflect' command to prevent the loss of atoms through the fixed boundary. The dimensions of the domain are 300 × 45 × 5 unit cells in the x,y, and z directions, respectively. This ensures the droplets can spread sufficiently without interacting across the x periodic boundary. The wall size in the y direction is set to 3 unit cells, which ensures a thickness larger than the interaction cut-off distance (r_{cut}). The droplets contain approximately 10,000 atoms.

The system is equilibrated under the micro-canonical ensemble at 100 K for 250 ns. The spreading of the droplets is assessed by tracking their center of mass. Following equilibration, the two-dimensional density profiles of the droplets are sampled for 50 ns. Subsequently, a circular fit is made through the droplet interface. This is identified by locating the "equimolar" points, where the local density is equal to the average of the bulk liquid and bulk vapour densities.[5] The angle that the circular fit through equimolar points makes with the wall is the resulting contact angle θ, as depicted in Figure S2. The resulting values of θ for several magnitudes of ε_{SL} can be seen in Table 1 of the main text.

3 Validation of G

As reported in the main article, our calculated values of G at 50 MPa agree very well with those of Sääskilahti et al.[1] for the same pressure at the three wettabilities (5 meV, 10 meV, and 50 meV) they

investigated, as shown in Figure S3. However, as Sääskilahti et al.[1] provided data solely for these wettabilities, they did not observe the cross-over in G.

Xue et al.[4] were the first to observe a transition in G from an exponential regime to a linear one for a Lennard-Jones (LJ) system. Modelling alternating slabs of solid and liquid atoms each 10 unit cells wide, for a total of four solid slabs and four liquid slabs, they observed the cross-over threshold to occur at $\varepsilon_{SL} = \varepsilon_{LL} = 10.3$ meV. Giri and Hopkins[3] also studied the relation between ε_{SL} and G at an LJ solid-liquid interface. However, they chose to simplify the set-up used by Xue et al.,[4] instead modelling a solid wall in the center of the domain encased by two liquid slabs to its left and right, with values of G reported in Figure S3. Giri and Hopkins[3] also mentioned the transition from an exponential regime to a linear one, but did not specify a cross-over threshold. In a subsequent publication, Giri et al.[2] utilised the same all-LJ system, reporting significantly different values of G (see Figure S3). It is unclear where these differences arise from as the parameters are identical in both setups. However, Giri et al.[2] observed the cross-over in G at the threshold of $\varepsilon_{SL} = \varepsilon_{LL} = 10.3$ meV, as originally discovered by Xue et al.[4] While an exact cross-over threshold was not reported in their earlier study,[3] it is clear from Figure S3 that this would be very different from their subsequent work. These differences are not explicitly acknowledged or addressed in Giri et al.[2]

Comparing our results to Giri et al.[2] in Figure S3, we find that our G values are lower in magnitude when $\varepsilon_{SL} < 10.3$ meV, and higher when $\varepsilon_{SL} > 10.3$ meV. This discrepancy makes our cross-over threshold further to the right at $\varepsilon_{SL} = 50$ meV instead of the threshold of $\varepsilon_{SL} = 10.3$ meV. It is important to note here that Giri et al.[2] equilibrated their system at 170K and 0 MPa, while we equilibrated our systems at 100K and 50 MPa. As a reduction in pressure would produce a decrease and not an increase in G,[1,6] the higher G values obtained by them for $\varepsilon_{SL} < 10.3$ meV cannot be attributed to a different system pressure.

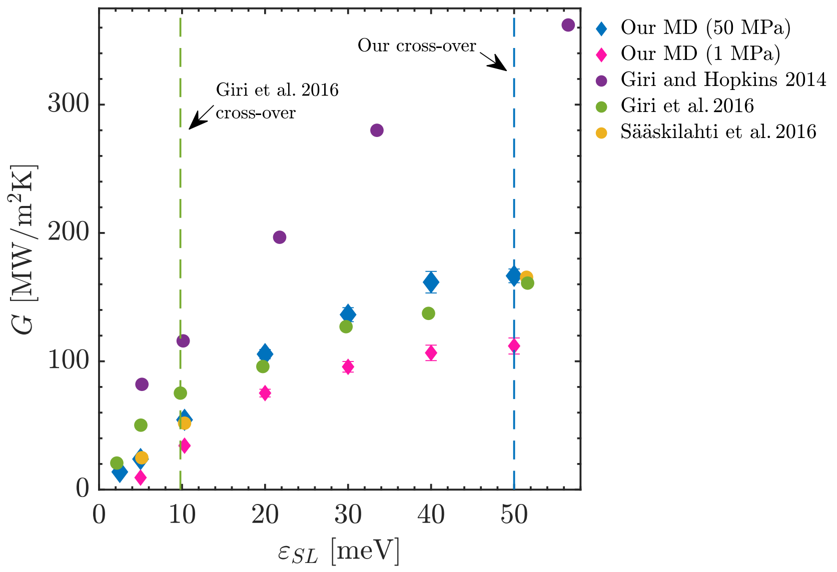

Figure S3: Dependence of interfacial thermal conductance (G) on solid/liquid interaction strength ε_{SL}. Our MD results (50 MPa) are compared to the values of G computed by Giri and Hopkins,[3] Sääskilahti et al.,[1] and Giri et al.[2] for similar Lennard-Jones (LJ) systems. Excellent agreement is found with results by Sääskilahti et al.[1]

In an attempt to replicate the results by Giri et al.,[2] we equilibrate our system at 1 MPa and compute G for the same range of ε_{SL}, as presented in Figure S3. We did not equilibrate the system at 0 MPa because the low magnitudes of G and our fixed heat-flux condition would cause the temperature of the interfacial fluid to exceed its saturation temperature, resulting in phase change. The additional 1 MPa of pressure ensures that phase change does not occur, while still having minimal influence on the magnitude of G.[6] While G values vary due to the reduction in pressure from 50 MPa to 1 MPa, we find that we still cannot reproduce the values of G and the cross-over threshold obtained by Giri et al.[2] Our values of G are still significantly lower than those reported by Giri et al.[2] across the entire range of wettabilities studied. Given our excellent agreement with Sääskilahti et al.[1] and the unusual choice of system pressure by Giri et al.,[2] we speculate that the pressure control scheme implemented by Giri et al.[2] could have yielded inconsistent pressures across the different values of

ε_{SL}. Additionally, as noted earlier, the LJ material being modelled cannot exist in its liquid phase at the 170K at 0 MPa state[7] specified by Giri et al.[2] As such, without explicitly computing the actual pressure within their system at each wettability, their results cannot be replicated.

Considering the differences in the thermodynamic state with Giri et al.[2] and the discrepancies between their two studies, as well as the difference in geometry compared to the system modelled by Xue et al.,[4] we rely on our validation with Sääskilahti et al.[1] for our simulation setup.

4 Interfacial Liquid Density Layering

The density layering was computed in the interfacial liquid by dividing it into rectangular bins of width 0.125 Å along the x axis (i.e. direction of heat flow, see Figure 1 of the main article) and computing the mass density ρ within each bin. The density layering is shown for select values of ε_{SL} in Figure S4. We observe increasing peak ρ with increasing ε_{SL}, consistent with literature.[8,9] We observe that full adhesion of the first liquid layer occurs around $\varepsilon_{SL} = 30$ meV, meaning that liquid density drops to zero in the first valley between adsorbed liquid layers, as annotated in Figure S4. This finding demonstrates that full adhesion of the first liquid layer cannot fully explain the cross-over in G, as it occurs prior to the cross-over threshold of $\varepsilon_{SL} = 50$ meV. Full adhesion of the second liquid layer does not occur even when $\varepsilon_{SL} = 100$ meV (ρ does not fall to zero in the second valley, see the inset of Figure S4), and again cannot be directly linked to the cross-over threshold. Note that the pronounced density layering and large magnitudes of peak ρ are a consequence of our chosen values for ε_{SL}.

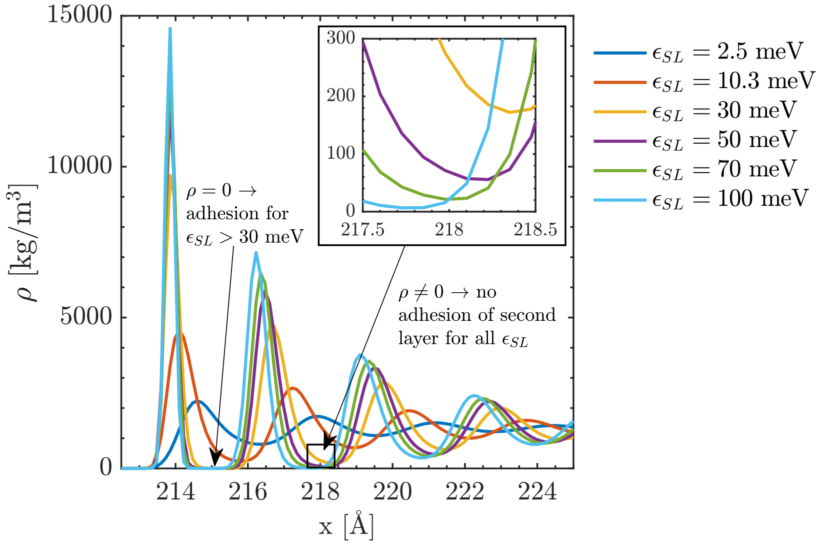

Figure S4: The mass density (ρ) of the interfacial liquid computed at select values of the solid/liquid interaction strength ε_{SL}. The inset shows that $\rho \neq 0$ in the valley of the second liquid layer for all ε_{SL}, demonstrating the lack of adhesion of the second layer for all wettabilities.

5 Interfacial Liquid Radial Distribution Functions

To further investigate if interfacial liquid structuring is linked to the cross-over, the radial distribution function (RDF) was computed within the first, second, and third interfacial liquid layers, as presented in Figure S5. We observe the onset of long-range ordering in layers 1 and 2 around $\varepsilon_{SL} = 30$ meV, as demonstrated by the presence of multiple peaks within 15 Å, as seen in Figure S5. As this occurs prior to the cross-over threshold, we conclude that in-plane structuring cannot fully explain the cross-over. No long-range ordering can be seen in layer 3, as demonstrated by the lack of further peaks beyond the first solvation shell across all wettabilities. Note that the significant degree of long-range ordering in layers 1 and 2 is a consequence of our chosen range of values for ε_{SL}.

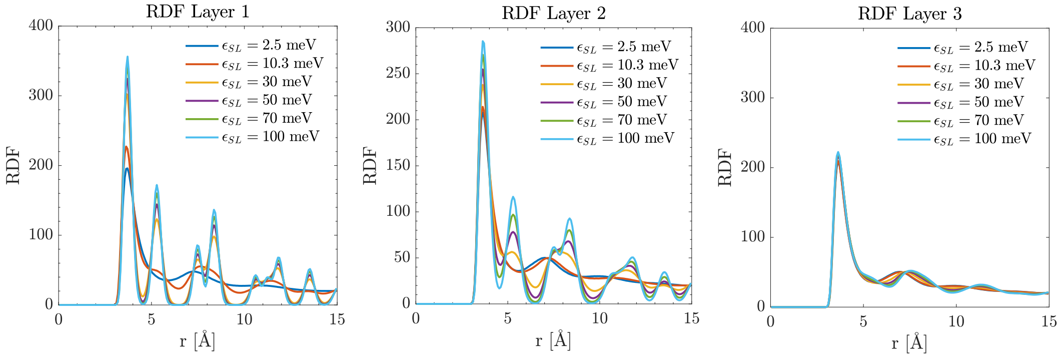

Figure S5: Radial Distribution Function (RDF) computed for the first, second, and third interfacial liquid layers.

6 Spectral Decomposition of Heat-flux

6.1 Methodology

The spectral decomposition of heat-flux (SDHF) is defined as the Fourier Transform of the force-velocity cross-correlation function (FVCF).[1] To bypass the time-consuming computation of the FVCF, the computational efficiency of the Fourier Transform can be exploited. The cross-correlation theorem states that the Fourier Transform of a cross-correlation function such as the FVCF is equivalent to the dot product between the complex conjugate of the Fourier transform of one time series and the Fourier Transform of the second time series.[10]

Using the cross-correlation theorem, the SDHF of the interfacial solid can be computed via:

$$\text{SDHF}_{solid}(\omega) = \frac{2}{AM}\Delta t_s \Re \sum_{j\in L}\sum_{i\in S}\langle F^*_{ij}(\omega)\cdot V_i(\omega)\rangle, \tag{1}$$

where A is the system's cross-sectional area, M is the length of the time series (i.e. number of samples), and Δt_s is the sampling interval. $\Re$ represents the real part of the resulting dot product. $F^*_{ij}(\omega)$ is the complex conjugate of the Fourier Transform of $F_{ij}(t)$, where $F_{ij}(t)$ is the time series of the cumulative force on a solid atom i due to all liquid atoms j within the cut-off distance. $V_i(\omega)$ represents the Fourier Transform of the velocity time series $v_i(t)$ of the solid atoms i.

To compute the SDHF of the interfacial liquid, we similarly use:

$$\text{SDHF}_{liquid}(\omega) = \frac{2}{AM}\Delta t_s \Re \sum_{j\in S}\sum_{i\in L}\langle F^*_{ij}(\omega)\cdot V_i(\omega)\rangle. \tag{2}$$

Here, $F^*_{ij}(\omega)$ remains the complex conjugate of the Fourier Transform of $F_{ij}(t)$. However, $F_{ij}(t)$ is now the time series of the cumulative force on a liquid atom i due to all solid atoms j within the cut-off distance. Similarly, $V_i(\omega)$ is now the Fourier transform of the velocity time series $v_i(t)$ of the liquid atoms i.

We compute the SDHF of both the interfacial solid and liquid by dividing the simulation into sequential time segments. Each time segment comprises 20,000 samples ($M = 20000$), sampled every 25 timesteps, with a timestep of 0.002 ps ($\Delta t_s = 25 \times 0.002 = 0.05$ ps). This yields time segments each 1 ns long. The SDHF of both media are computed from the independent time segments, and are then averaged to yield the spectra presented.

6.2 Validation

To validate our SDHF calculation methodology, we replicate the system simulated by Sääskilahti et al.[1] and compute the SDHF of the interfacial solid. Dividing the SDHF by the interfacial temperature discontinuity ΔT yields the spectral decomposition of the interfacial conductance. This is then plotted against the results presented by Sääskilahti et al.[1] Reasonable agreement can be seen in Figure S6, demonstrating the validity of our approach. Statistical noise in SDHF predictions can be diminished by computing more time windows and further averaging.

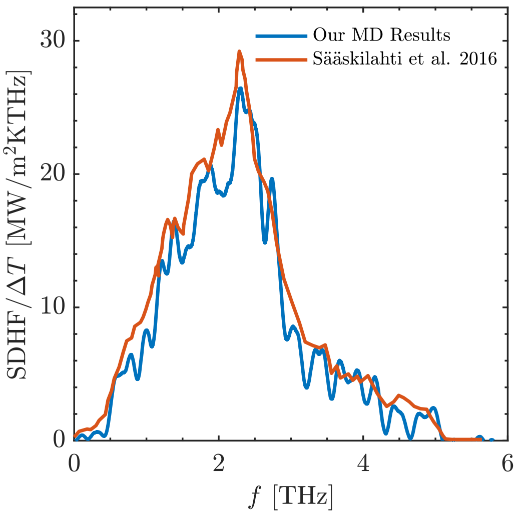

Figure S6: The spectral decomposition of G at the interfacial solid, defined as SDHF(ω)/ΔT, computed for the solid/liquid interaction strength ε_{SL} = 10.3 meV. Reasonable agreement can be seen when comparing to results by Sääskilahti et al. [1] for the same system.

7 Vibrational Density of States

7.1 Methodology

The vibrational density of states (VDOS) is computed from the Fourier transform of the velocity autocorrelation function (VACF) for a group of atoms, where the time-varying VACF is defined as [11]

$$\text{VACF}(t) = \langle v_i(t) \cdot v_i(0) \rangle. \tag{3}$$

Here, $v_i(t)$ represents the velocity of an atom i at time t, while $v_i(0)$ represents the initial velocity. Similar to the computation of the SDHF, the computational efficiency of the Fourier Transform can be made use of to avoid the direct computation of the VACF. The autocorrelation theorem is a specific case of the cross-correlation theorem where the two time series are the same, and states that the Fourier Transform of an autocorrelation function is equivalent to the squared modulus of the Fourier Transform of the original time series. [12] Thus, the VDOS can be obtained using:

$$\text{VDOS}(\omega) = \sum_{i=0}^{N} \left| \int_0^\infty v_i(t) e^{-i\omega t} \right|^2, \tag{4}$$

where i is the atom belonging to a group of atoms of size N.

In the case of the LJ solid, we compute the interfacial VDOS from the outermost layer of the solid in contact with the liquid. For the LJ fluid, we compute the interfacial VDOS from the first adhered liquid layer, defined as first peak in the interfacial liquid density layering, as presented in Section 3. To account for the diffusion of the liquid, we only compute the VDOS for atoms that remain within the interfacial region for the entirety of the calculation, discarding atoms that diffuse in/out of this region. [13] Similar to the computation of the SDHF, we divide the simulation into sequential time segments. Each segment is 500 samples long ($M = 500$), sampled at intervals of 25 timesteps. For a timestep of 0.002 ps, this results in time segments each 25 ps long. The resulting spectra are then averaged to yield the VDOS presented.

7.2 Validation

To validate our methodology, we compute the VDOS of bulk aluminium at 300K and validate our normalised results against those reported by Korotaev et al. [14] Excellent agreement can be seen in Figure S7, thus validating our VDOS calculations.

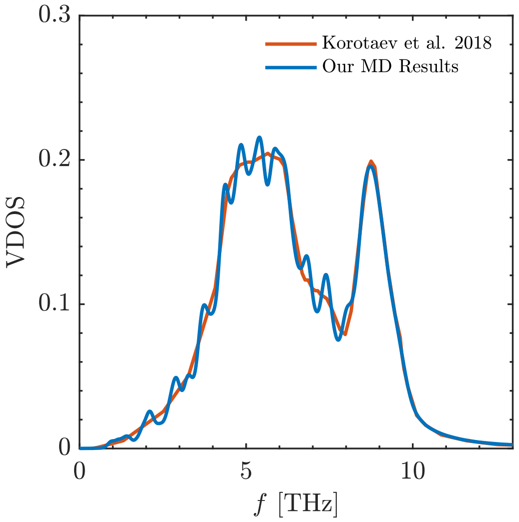

Figure S7: The vibrational density of states (VDOS) of bulk aluminium at 300K. Excellent agreement can be seen when comparing to results by Korotaev et al. [14]

References

[1] K. Sääskilahti, J. Oksanen, J. Tulkki, and S. Volz. Spectral mapping of heat transfer mechanisms at liquid-solid interfaces. *Physical Review E*, 93(5):1–8, 2016.

[2] Ashutosh Giri, Jeffrey L. Braun, and Patrick E. Hopkins. Implications of interfacial bond strength on the spectral contributions to thermal boundary conductance across solid, liquid, and gas interfaces: A molecular dynamics study. *Journal of Physical Chemistry C*, 120(43): 24847–24856, 2016.

[3] Ashutosh Giri and Patrick E. Hopkins. Spectral analysis of thermal boundary conductance across solid/classical liquid interfaces: A molecular dynamics study. *Applied Physics Letters*, 105(3):033106, 2014.

[4] L. Xue, P. Keblinski, S. R. Phillpot, S. U.S. Choi, and J. A. Eastman. Two regimes of thermal resistance at a liquid-solid interface. *Journal of Chemical Physics*, 118(1):337–339, 2003.

[5] Sreehari Perumanath, Mykyta V. Chubynsky, Rohit Pillai, Matthew K. Borg, and James E. Sprittles. Rolling and Sliding Modes of Nanodroplet Spreading: Molecular Simulations and a Continuum Approach. *Physical Review Letters*, 131(16):164001, 2023.

[6] Khaled Issa and Abdulmajeed Mohamad. Pressure Effects on Liquid-solid Interfacial Thermal Resistance. *3rd International Conference on Nanotechnology: Fundamentals and Applications (ICNFA)*, (332):7–9, 2012.

[7] AL Grossman, Jerome G Hust, and Robert D Mc Carty. Thermodynamic properties of argon from the triple point to 300 k at pressures to 1000 atmospheres. Technical report, 1969.

[8] Dmitry Alexeev, Jie Chen, Jens H. Walther, Konstantinos P. Giapis, Panagiotis Angelikopoulos, and Petros Koumoutsakos. Kapitza Resistance between Few-Layer Graphene and Water: Liquid Layering Effects. *Nano Letters*, 15(9):5744–5749, 2015.

[9] Yali Ma, Zhongwei Zhang, Jige Chen, Kimmo Sääskilahti, Sebastian Volz, and Jie Chen. Ordered water layers by interfacial charge decoration leading to an ultra-low Kapitza resistance between graphene and water. *Carbon*, 135:263–269, 2018.

[10] Fred J. Taylor. Signal processing, digital. In Robert A. Meyers, editor, *Encyclopedia of Physical Science and Technology (Third Edition)*, pages 737–760. Academic Press, New York, third edition edition, 2003.

[11] Michael P Allen and Dominic J Tildesley. *Computer simulation of liquids*. Oxford university press, 2017.

[12] Ronald N. Bracewell. *The Fourier transform and its applications*. McGraw-Hill Higher Education, 3rd edition. edition, 2000.

[13] Juan D. Olarte-Plata and Fernando Bresme. Thermal conductance of the water-gold interface: The impact of the treatment of surface polarization in non-equilibrium molecular simulations. *Journal of Chemical Physics*, 156(20):204701, 2022.

[14] Pavel Korotaev, Maxim Belov, and Aleksey Yanilkin. Reproducibility of vibrational free energy by different methods. *Computational Materials Science*, 150(February):47–53, 2018.
